# How Long Does Craving Predict Use of Methamphetamine? Assessment of Use One to Seven Weeks After the Assessment of Craving

**Published:** 2008-08-26

**Authors:** Gantt P. Galloway, Edward G. Singleton, The Methamphetamine Treatment Project Corporate Authors

**Affiliations:** 1 Addiction Pharmacology Research Laboratory, St. Lukes Hospital, 7th floor, 3555 Cesar Chavez Street, San Francisco, CA 94110.; 2 The MayaTech Corporation, 1100 Wayne Avenue, Suite 900, Silver Spring, MD 20910.

**Keywords:** methamphetamine, craving, relapse, predictive validity

## Abstract

**Aims:**

This study lays the foundation for a clinical prediction model based on methamphetamine craving intensity and its ability to predict the presence or absence of within-treatment methamphetamine use.

**Design:**

We used a random effects logistic approach for estimating repeated-measures, generalized linear mixed models (GLMM) using craving as the sole predictor of methamphetamine. A multivariate GLMM included craving, length of treatment, treatment assignment, and methamphetamine use the previous week as covariates to control for potential confounds. We performed receiver operating characteristic (ROC) analyses to evaluate predictive accuracy. We investigated further whether methamphetamine craving predicted subsequent use more accurately at intervals more proximal to versus those more distal to assessment, examining one-week periods ending one to seven weeks after assessment of craving.

**Setting:**

The study was part of the Center for Substance Abuse Treatment (CSAT) Methamphetamine Treatment Project (MTP).

**Subjects:**

Analyses were based on data from 691 methamphetamine dependent outpatients enrolled in the MTP.

**Measurements:**

Craving was assessed by self-report on a 0–100 scale. Self-reported methamphetamine use was toxicologically verified. Craving and drug use were assessed weekly for 8 weeks.

**Findings:**

In the univariate analysis craving predicted methamphetamine use in the week immediately following the craving report (p < 0.0001), with subject-specific use increasing 0.38% for each one-point increase in craving on a 0–100 scale. In the multivariate analysis the probability of use decreased by 2.45% for each week in treatment increased by 33.11% for previous methamphetamine use, and the probability of methamphetamine use still increased with craving, rising 0.28% for each one-point increase in craving score (all p < 0.0001). Predictive accuracy was strongest at the one-week time-lag and declined in magnitude the more distal the assessment period.

**Conclusions:**

Craving is a predictor of within-treatment methamphetamine use. Intensity of craving is appropriate for use as a surrogate marker in methamphetamine dependence.

## Introduction

### Craving as a target for treatment intervention

Several interpretations of addiction propose that craving is a primary motivation for drug use and a principal contributor to relapse [1–9]. For example, drug craving is largely characterized by obsessive thinking about drugs, triggering compulsive drug-seeking and subsequent drug use [3]. Robinson and Berridge [2] state similarly that “ … addicts develop an obsessive craving for drugs, a craving so irresistible that it almost inevitably leads to drug seeking and drug taking … drug craving is fundamental to addiction”. Craving is experienced by most persons during withdrawal, a central feature of DSM-IV substance-induced disorders [10]. Craving also is a key element of substance dependence [11–13] and is included as an optional diagnostic criterion for addiction in the International Classification of Diseases (ICD-10; [14]). Thus, drug craving has become an appropriate target for treatment intervention [15–17] and relapse prevention [18], particularly with respect to abstinence from ongoing drug use despite high levels of craving or the growing and irresistible urge to use drugs.

Notwithstanding the important role that drug craving plays in research and clinical settings, there is uncertainty whether it actually drives drug use. Experimental manipulations of craving have been successfully modelled in research settings, yet cocaine craving did not influence immediate cocaine-seeking behavior under laboratory conditions [19], though acute abstinence effects such as craving may be minimal [20]. Tobacco craving has been reliably suppressed by aversive rapid smoking in a laboratory setting, yet the intensity of craving scores did not affect immediate smoking [21]. Self-reported cue-induced craving obtained in the laboratory also rarely correlates with subsequent real-life relapse [22, 23], although cue-reactivity trials have demonstrated that alcohol craving in the laboratory was modestly correlated with alcohol craving in the field, which was significantly correlated with real-life drinking [24].

Whether craving is a determinant of drug use, however, is not the only factor in whether it is an appropriate intervention target. No doubt, craving is a contributor to processes involved in addiction such as progression to compulsive use, dependence, or relapse. Craving is frequently referenced as a proxy measure of the probability of drug use [25, 26] and the most commonly targeted clinical endpoint is ongoing drug use [27, 28]. To be a reliable surrogate [29], craving must track consistently with the presence or absence of drug use. Though alleviation of craving is sometimes expected to reduce the risk of ongoing drug use, it is not absolutely essential that its reduction halts the progression of addictive processes. It is essential, however, to establish craving as a risk marker sufficiently distal from the endpoint to provide time to intervene and prevent ongoing drug use (not necessarily target craving), which in turn is expected to disrupt the progression of compulsive use, dependence, and relapse. The critical issue remains whether craving is a valid predictor of the probability of ongoing drug use [25].

### The relevance of craving has been questioned

Inconsistent findings regarding the ability of craving to track ongoing drug use, have led to doubts about its predictive validity. For example, craving significantly predicted six-month relapse to drinking in an early study [30], though it did not predict alcohol and drug use two years following substance abuse treatment in a later study by Walton and colleagues [31]. Neither the Yale-Brown Obsessive Compulsive Scale for heavy drinking [32], a rating of the severity of obsessive-compulsive symptoms, nor a single item visualanalogue craving scale (VAS) was able to predict either complete abstinence or the number of days abstinent from alcohol use [33], and other studies using the total score for craving-related OCS on the Obsessive Compulsive Drinking Scale [34] showed limited predictive validity for subsequent drinking at post treatment follow-up [35, 36]. However, research using the Penn Alcohol Craving Scale found significant differences in craving scores during the initial three weeks of treatment among subjects that did and did not relapse to drinking during treatment weeks three to twelve [37]. With respect to cue-induced craving, higher levels of alcohol craving in response to drinking role-plays predicted increased alcohol use six months post treatment, however, craving in reaction to beverage cues was inconsistently predictive of outcome [38]. Another study [39] had shown that craving might actually protect some drinkers against ongoing alcohol use.

Other studies also questioned the validity of craving as a reliable predictor of ongoing drug use [40–45]. For example, in the context of opioid and cocaine dependence, baseline heroin craving was not predictive of ongoing heroin use during a 21-day outpatient medically supervised detoxification, though baseline scores on two measures of cocaine craving were significant predictors of in-treatment cocaine use [45]. Yet, cocaine craving at baseline was not predictive of relapse when abstinence was assessed in the last two weeks of eight weeks of outpatient treatment [41], and was uncorrelated with sustained abstinence one year after craving was assessed [40]. Similarly, cocaine craving was not predictive of relapse in 35 cocaine dependent inpatients when use was assessed three months after discharge [42].

Nevertheless, limitations in design may have contributed to the inconsistencies, including the use of retrospective self-reports, insufficient statistical power, and the time lag between assessment and endpoint [46]. For one thing, some studies used retrospective reports following relapse [47, 48], which may be inaccurate and biased by exaggerated negative affect [49, 50]. In general, retrospective studies challenged the belief that craving is significantly associated with ongoing drug use [4, 48, 51]. In one influential report, subjects were contacted one year after residential treatment for substance abuse and asked the cause of their first relapse [48]. Only seven percent attributed their first relapse to craving; impulsive action was the most common (21%) self-reported cause. Ludwig et al. [52] reported that, following treatment, only one percent of subjects attributed their relapse to craving, and more recently, only five percent of those who relapsed during treatment cited craving as the primary reason for their alcohol or other drug use [4].

### Design and sample size determine whether craving predicts drug use

In sharp contrast to the studies reviewed above, a series of prospective studies have found craving to be highly predictive of the presence or absence of ongoing smoking [53–55]. Baseline craving intensity was a significant and independent predictor of abstinence for 11–52 weeks in a recent randomized clinical trial of the efficacy of sustained release bupropion [55]. In 2,645 nicotine dependent outpatients, craving was assessed 24 to 48 hours after smoking cessation and smoking status was assessed one year later. Overall, 16% of those with immediate post-cessation craving scores in the highest quartile remained abstinent at the 12-month follow-up compared with 31% in the lowest quartile. Furthermore, more than 32% of those with high craving scores relapsed within one week of smoking cessation [54]. In a similar study, Killen et al. [53] had reported that only 26% of those with high initial craving remained abstinent at two-month follow-up. Other research [56] also found that craving predicts ongoing smoking at 10- and 15-week follow-ups.

Because of the small number of observations used in the analyses, several studies may have lacked sufficient statistical power to detect significant results [19–21, 35, 36, 45]. As noted by Shiffman and colleagues [57], longitudinal studies should concentrate on within-subject variation, and take advantage of repeated measurements. Investigations utilizing those methods have demonstrated that ongoing cigarette use was associated with real-life exposure to smoking-associated cues [57, 58]. Another design issue involves the time lag between self-reported craving and the subsequent assessment of alcohol or other drug use [59]. Many researchers assessed drug use several months after craving was measured [40–43]. Such assessment periods are appropriate when craving scores are used solely as an outcome measure (craving reduction as treatment success), but shorter intervals may be appropriate to test the hypothesis that craving is predictive of subsequent drug use. For example, cocaine craving significantly predicted drug-use outcomes during treatment independently of the pretreatment quantity of cocaine use [17]. Craving also significantly predicted relapse to drinking, with more proximal assessment (within a prior eight-week period) substantially improving predictive power [30]. In addition, Killen and others [53, 54] have shown that ongoing tobacco use for high cravers occurred rapidly, often within a one week period following craving assessment.

The few studies that have addressed these design limitations have consistently yielded significant relationships between craving and drug use. For example, Flannery et al. [60] used generalized estimating equations (GEE; [61]) methodology to determine whether three craving instruments could successfully predict drinking during treatment. Craving was assessed weekly or bi-weekly during a nine-month, double-blind placebo-controlled trial of naltrexone and psychosocial intervention. Each of the three instruments used to assess craving significantly predicted drinking in the subsequent treatment week. Interestingly, craving was a stronger predictor of subsequent drinking than was drinking during the prior week. Using GEE methodology in a 24-week trial of 449 cocaine dependent outpatients, a higher score on a three-item Cocaine Craving Scale was a statistically significant predictor of cocaine use in the subsequent treatment week; each one-point increase on the composite score of the craving scale was associated with a 10% increase in the risk of using cocaine in the next week [62]. However, among patients who received individual plus group drug counseling, the treatment condition with the best overall cocaine use outcome, increased craving scores were not associated with greater likelihood of cocaine use in the subsequent treatment week.

It is important to note that GEE methodology yields population-based analyses [63], which are useful in outcome evaluations (e.g. within-subjects designs with more than one treatment; extended pre-post [panel data] designs) and in clinical trials or comparative studies for helping identify pharmacological and nonpharmacological interventions with possible therapeutic value for specific patient groups. What is missing from the argument, however, is that craving should be a “good” risk (prognostic) marker of the probability of ongoing use for a specific patient [29], apart from the particular treatment that patient receives, the length of intervention, or a history of prior drug use.

### Previous work examining the role of craving as a predictor of methamphetamine use

Hartz and colleagues [46] were the first group to examine the role of craving in predicting the probability of subject-specific drug use in a prospective, repeated-measure, within-subject analysis using a time-lagged design. Thirty-one individuals in treatment for methamphetamine dependence were asked to indicate once each week for 12 weeks the intensity of craving they had experienced during the previous 24 hours using a 100-mm VAS. Methamphetamine use was self-reported at each visit and toxicologically verified. A repeated measures, generalized linear mixed model (GLMM) was fit to binary outcomes (use and nonuse) indicating correct verification. Methamphetamine craving significantly predicted methamphetamine use in the week immediately following each craving assessment, such that for the typical subject the probability of use in the subsequent treatment week increased by 0.35% for each one-point increase in craving score. Using a median-split cut-off point, the relative risk of subsequent use was also 2.1 times greater for craving scores in the upper half compared with scores in the lower half. Furthermore, craving scores preceding use were 2.7 times higher than scores that preceded nonuse. Craving also remained a highly significant predictor in multivariate GLMM models after controlling both for pharmacological intervention and for methamphetamine use during the previous week. Findings have promising therapeutic implications for craving as a risk marker, given that a one week time lag is sufficiently distal from the endpoint to identify a patient at greater risk of ongoing drug use that could benefit from a more tailored treatment strategy.

Thus, the purpose of this study is to set the foundation for a clinical prediction model based on methamphetamine craving intensity and its ability to track the presence or absence of within-treatment methamphetamine use. Reproducibility is essential but lacking in the literature, thus we replicated first the findings of Hartz et al. [46] to determine if methamphetamine craving is a valid and reliable predictor of the probability of ongoing methamphetamine use. Another issue is the strength of prediction [25] or the ability of the craving to distinguish correctly patients that do from those that do not use. Following Hughes’ recommendations, we derived next sensitivity, specificity, and other indices of predictive accuracy to determine whether craving is a potential prognostic marker for subject-specific methamphetamine use. In the absence of systematic research examining the time-lag between craving and drug use endpoints, we investigated further whether the intensity of self-reported methamphetamine craving predicted subsequent methamphetamine use more accurately at intervals more proximal to assessment of use compared to those more distal to assessment of use, examining biochemically-verified end points ranging from one to seven weeks after the craving assessment.

## Methods

### Study Design

The present study was part of the Center for Substance Abuse Treatment (CSAT) Methamphetamine Treatment Project (MTP), the largest randomized clinical trial of behavioral treatments for methamphetamine dependence [64]. MTP was conducted in eight community outpatient settings: the coordinating center was at the University of California, Los Angeles (UCLA), while the other seven investigative teams conducted the study at eight sites in Northern and Southern California, Hawaii, and Montana. Subjects were randomly assigned to receive either the manualized Matrix Model or treatment-as-usual (TAU) at each site [64–67]. Research assistants at each site were trained and certified in standard operating procedures, data collection, and instrument administration. Craving and drug use were assessed once weekly. Subjects gave written informed consent according to guidelines for the protection of human research volunteers of the U.S. Department of Health and Human Services. Each local Institutional Review Board approved the study.

### Subjects

Subjects (n = 691) were recruited by advertisement, referrals from community agencies (medical, substance abuse, mental health, and criminal justice) and by word of mouth. Subjects were included if they were at least 18 years of age, methamphetamine dependent per DSM-IV criteria, willing to complete forms and provide urine samples, provide informed consent, able to understand scales and instructions, able to understand English, and participate in all aspects of either treatment condition. Subjects were excluded if they had not used methamphetamine in the past 30 days (unless in a controlled environment such as jail or prison in which case the requirement was methamphetamine use in the past 45 days), required medical detoxification from opioids, alcohol and other drugs, were enrolled in another treatment program in the past 30 days, or had medical, legal, housing, or transportation issues precluding safe and consistent participation.

Data was based on a study population of 691 subjects. Craving data was available for 864 subjects enrolled in the MTP. Because of the time-lagged nature of the design, subjects had to contribute at least one craving-methamphetamine use lagged pair. We focused on the eight week time frame that is common to clinical trials. Observations from 11 subjects were excluded because they did not return for a visit until 8–16 weeks subsequent to the initial assessment. Observations from 114 subjects were also excluded because they attended only once and 48 subjects could only contribute craving-methamphetamine use lagged pairs for weeks 2–7.

In a preliminary investigation, no significant differences in drug use and functioning had been found between Matrix and TAU subjects, at either discharge or six-month follow-up [64]. Matrix Model subjects, however, had better treatment retention and completion rates, and were more likely to have methamphetamine-free urine test results while in treatment compared to TAU subjects. In this study, characteristics of the subject population (see Table 1) were consistent with clinical treatment samples studied previously [68, 69]. The majority of the subjects were female (52%) and Caucasian (63%). Self-reports and urinalysis confirmed that subjects most often used methamphetamine, marijuana, and alcohol throughout the duration of the study. The subjects had on average five years of frequent or problematic methamphetamine use, and 12 days of methamphetamine use in the past 30 days. Smoking was the usual route of methamphetamine administration for most (61%) of the subjects.

### Measures

Subjects self-reported the most severe craving experience on the previous day on a 0–100 scale. Endpoints anchors were “no craving” and “most craving ever experienced”. Craving was defined as “an urgent desire, longing, or yearning, not just a passing thought”. Self-report of methamphetamine use was assessed by eliciting reports of the number of days of use since the previous assessment. Urine samples were tested weekly for methamphetamine by immunoassay screening and gas chromatography/mass spectroscopy (GC/MS) confirmation and quantification. Weeks were classified as non-use weeks if no methamphetamine was used per self-report and the urinalysis was negative for methamphetamine; weeks were classified as use weeks if the self-report indicated methamphetamine use or if the GC/MS result was greater than or equal to 1,000 ng/ml. Craving and drug use were assessed once weekly.

### Data Analyses

**Figure 1:**
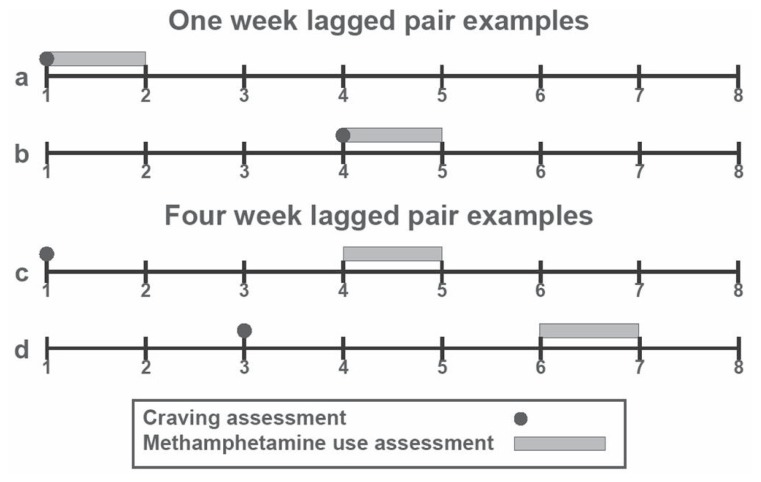
Examples of craving-methamphetamine use lagged pairs.

Following Hartz et al. [46], we used a random effects logistic approach for estimating the clinical prediction model based on methamphetamine craving intensity and its ability to track the presence or absence of ongoing methamphetamine use. Consistent with previous research on more proximal assessment of rapid return to ongoing drug use [30, 53, 54], we restricted the analysis to an eight-week assessment period. Figure 1 depicts examples of the time-lag from craving assessment to methamphetamine use or abstinence (endpoint). We estimated first a univariate, repeated-measures, generalized linear mixed model [70] with craving as the sole predictor of methamphetamine use (coded positive [presence] and negative [absence]) during the week immediately following each craving assessment (i.e. week one craving was paired with week two methamphetamine use, week two was paired with week three methamphetamine use, …, and week seven was paired with week eight methamphetamine use; see Figure 1, 1a and 1b). Each subject could therefore contribute up to seven lagged pairs to the analysis. Total observations consisted of 2,742 time-lagged data point pairs (approximately four observations per subject), which were randomly split (33%/67%) for the purpose of double cross-validation to guard against overfitting the data, estimate internal replicability, and reduce capitalization on chance [71, 72]. Models were fit using the GLIMMIX macro in SAS [73], specifying a binomial error structure and logit link function, with subjects as a random factor and craving as a fixed effect (predictor). We pooled observations from all sites and modeled length of treatment (weeks) as a random effect (quadratic trend) to account for unobserved (residual) heterogeneity. Next, we estimated three univariate, repeated-measure GLMMs, with either length of treatment, treatment assignment (TAU or Matrix Model), or biochemically-verified methamphetamine use the previous week as the only fixed effect. We also combined craving, length of treatment, treatment assignment, and biochemically-verified methamphetamine use the previous week in a multivariate GLMM model to control for potential confounds.

To evaluate strength of prediction, we used receiver operating characteristic (ROC) analyses and methods for estimating the area under the ROC curve (AUC) for correlated data (repeated-measure ROC; [74]). Next, optimal cut-off points for classifying positives (predicted use) and negatives (predicted nonuse) were derived by rounding the value yielding the maximum sum of sensitivity (Sn) and specificity (Sp) corresponding to the shoulder at the top left of the ROC curve. Then, we assessed predictive accuracy using several summary statistics [75], including the main outcome measures, AUC, Sn, Sp, and positive (LR+) and negative likelihood ratios (LR−). The rule-of-thumb for classification accuracy is AUC ranging 0.90–1 = excellent, 0.80–0.90 = good, 0.70–0.80 = fair, 0.60–0.70 = poor, and <0.60 = inadequate [76]. Conventional standards for sensitivity and specificity are 95% and 80%, respectively [77]. LR > 1 indicate increased probability of methamphetamine use, while LR < 1 indicate decreased probability of methamphetamine use, and general guidelines for interpreting likelihood ratios [78] are LR+ or LR- = 1, <2 or >0.5, 2–5 or 0.2–0.5, 5–10 or 0.1–0.2, and >10 or <0.1 correspond with no, minimal, small, moderate, and large increased or decreased risk, respectively. Likelihood ratios also are used to calculate predictive efficiency, the difference between pre-assessment and post-assessment probability of ongoing methamphetamine use, which is an effective measure of whether using risk marker can improve clinical decision-making for individual patients. For purposes of comparison with Hartz et al. [46], we also examined the relative risk of subsequent methamphetamine use for craving scores preceding use compared to those that preceded abstinence as well as for scores above vs. below the cut-off point. All summary statistics were derived from formulas identified in the literature [77–80], and calculations were performed using customized Excel spreadsheet software [81].

To examine the predictive accuracy of proximal vs. distal predictions, we estimated six additional GLMMs (one for each of the two to seven week time-lags) using craving as the sole predictor of ongoing methamphetamine use (i.e. week one craving was paired with week three methamphetamine use, week two craving was paired with week four methamphetamine use, …, week six craving was paired with week eight methamphetamine use [two weeks distal]; week one craving was paired with week four methamphetamine use, week two craving was paired with week five methamphetamine use, …, week five craving was paired with week eight methamphetamine use in [three weeks distal]; …; and week one craving was paired with week eight methamphetamine use [seven weeks distal]; see Figure 1, 1c and 1d). For comparative purposes, we also performed ROC analyses to estimate AUCs, derived optimal cut-off points, and calculated summary statistics for each of the six prediction models. To facilitate interpretation, this was followed by visual inspection of normalized trends produced by robust locally weighted regression [82] using SAS PROC LOWESS [83], which fit a line estimating the mean number of uses per assessment period as a function of the same range of craving scores (0–100) for each time lag (seven total).

## Results

### Internal and External Reproducibility

For the double cross-validation, the two-thirds sample of one-week time-lagged observations of methamphetamine use and non-use was predicted using the optimal cut-off point derived from the GLMM estimate for the one-third sample, then the one-third sample of one-week time-lagged observations of methamphetamine use and non-use was predicted using the optimal cut-off point derived from the GLMM estimate for the two-thirds sample. AUC was 0.70 and the optimal cut-off for the intensity level of methamphetamine craving was 27 regardless of which model was used to estimate them, therefore we combined observations. Overall use rate (prevalence) was 30% (813/2,742). The mean (SD) craving score was 17 (31), and 69% of reported values of craving were zero (1,902/2,742).

In the univariate analysis, methamphetamine craving significantly predicted methamphetamine use in the week immediately following each craving assessment (p < 0.0001). The probability of methamphetamine use for the average participant increased by 0.38% for each one-point increase in craving score. Relative risk of subsequent use also was 2.5 times greater for craving scores in the upper portion of the scale (RR = 0.52) relative to scores in the lower portion (cf. RR = 0.21) using this cut point. Similar to Hartz et al. [46] findings, craving scores preceding use (mean = 34.9) were 2.7 times higher (cf. 2.7 in Hartz et al.) than scores that preceded abstinence (cf. mean = 12.7). Univariate analyses also indicated that length of treatment and biochemically-verified methamphetamine use the previous week (both p < 0.0001), but not treatment assignment (p > 0.10), significantly predicted use, such that the probability of methamphetamine use in the week immediately following each craving assessment decreased by 2.45% for each week in treatment and increased by 33.11% for methamphetamine use the previous week. When controlling for length of treatment and methamphetamine use the previous week, the probability of methamphetamine use still increased significantly (p < 0.0001), rising 0.28% for each one-point increase in craving score.

### Predictive accuracy

Table 2 presents the summary statistics used to evaluate whether methamphetamine craving is a potential risk marker for ongoing methamphetamine use during treatment. At the one week endpoint in Hartz et al. [46], craving scores equaled or exceeded the median split cut-off point (marker predicted positive) for 122/243 observations and fell below the median split cut-off point (marker predicted positive) for 121/243 observations. The number of actual (biochemically verified) positives and negatives was 110 and 133, respectively, for methamphetamine use in the week immediately following each craving assessment. Two groups were classified correctly: predicted positive and actual positive (true positives; n = 75) and predicted negative and actual negative (true negatives; n = 87). Two groups were classified incorrectly: predicted positive but actual negative (false positives; n = 46) and predicted negative but actual positive (false negatives; n = 75).

The sensitivity (0.68) indicated that the levels of methamphetamine craving above the cut-off were accurate at identifying true positives more than two-thirds of the time, while the remaining 32% were false negatives, classified incorrectly as negative when in fact they were positive. Specificity, the proportion of true negatives correctly identified, was 65%, and the remaining 35% were false positives that were incorrectly classified as positive though they did not use methamphetamine in the week immediately after the craving assessment. The likelihood of predicting methamphetamine use in the subsequent treatment week increased 17%, rising from 0.45 (prevalence) to 0.62 (positive predictive value). The likelihood of predicting nonuse in the subsequent treatment week rose from 55% to 71% (negative predictive value), with a corresponding 16% decrease in the likelihood of actual use despite the marker predicting nonuse. Craving scores (marker) greater than or equal to the optimal were twice (positive likelihood ratio = 1.97) as likely to come from observations indicating actual use than those indicating nonuse.

Of 2,742 observations at the one-week endpoint in the current study (Table 2, column 1), 382 were true positives, 1579 were true negatives, 350 were false positives, and 431 were false negatives. Craving accurately detected 47% of the true positives, with the remaining 53% classified incorrectly as negative. The proportion of true negatives, however, was 82%; only 18% were incorrectly classified as positive. The likelihood of predicting methamphetamine use increased 22%, rising from 30% (use prevalence) to 52% (positive predictive value). While the likelihood of predicting nonuse increased from 70% (nonuse prevalence) to 79% (negative predictive value), such that there was a corresponding 9% decrease in the likelihood of actual use despite the marker predicting nonuse in the subsequent treatment week. Marker results at or exceeding the optimal cutoff were nearly three (positive likelihood ratio = 2.59) times more likely to result in use than nonuse.

### Proximal vs distal assessment

Table 2 also presents the summary statistics used to evaluate whether craving predicted methamphetamine use more accurately during weeks that were more proximal to the assessment of craving compared to those that were more distal to the assessment of craving (Table 2, columns 1–7). GLMM indicated that methamphetamine craving significantly predicted methamphetamine use across all time lags (all p < 0.036; not shown); however, AUCs declined in magnitude the more distal the assessment period. Optimizing cut-off point values also did not offset the decline in AUCs the more distal the week for which use was predicted.

**Figure 2:**
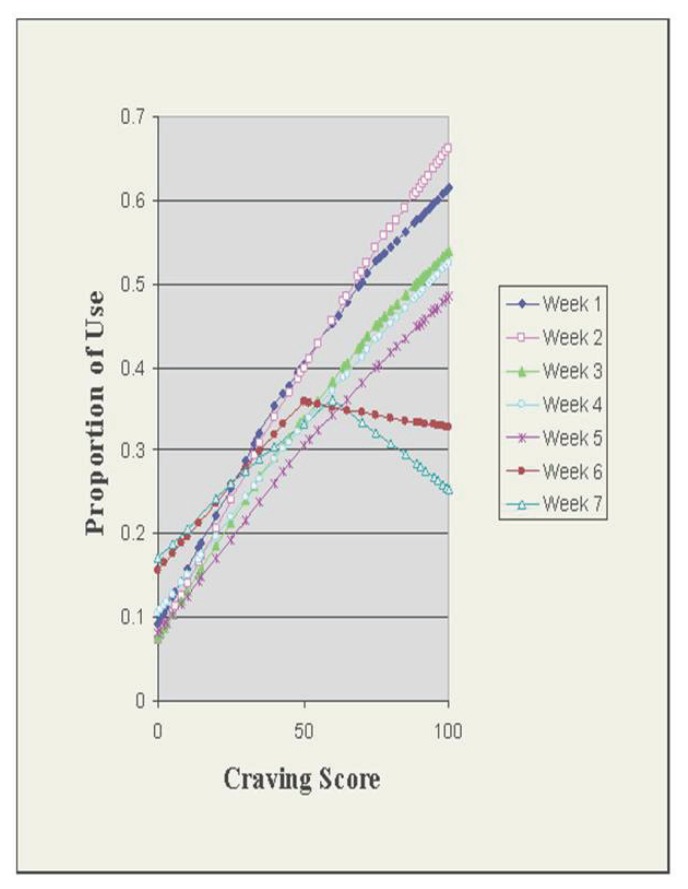
LOWESS analysis of craving vs methamphetamine use.

**Figure 3:**
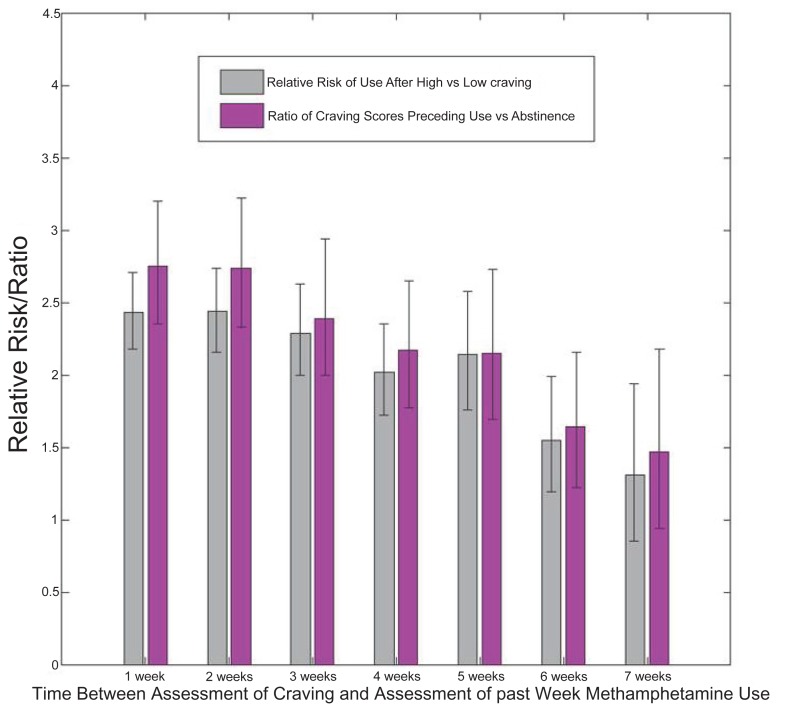
Relationships between craving and subsequent methamphetamine use.

**Figure 4:**
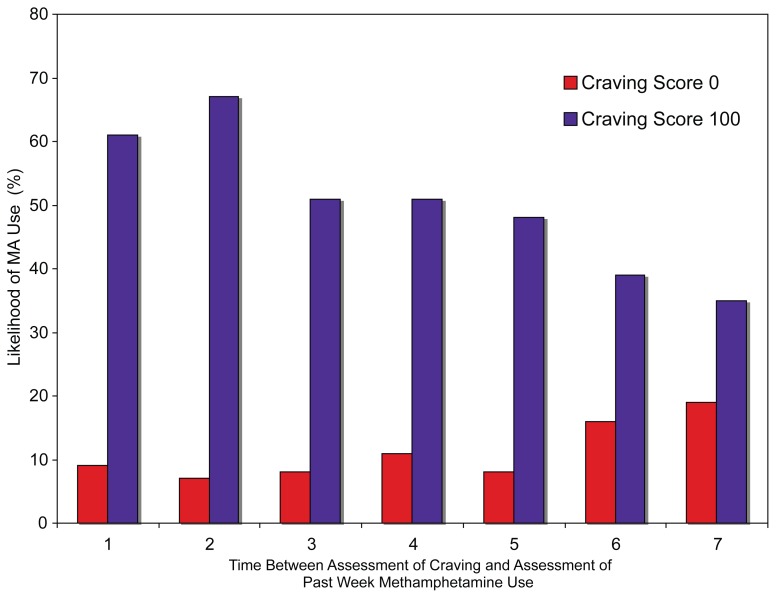
Probability of use of methamphetamine after minimal and maximal craving scores.

Predictions based on normalization using LOWESS curves exhibited similar declines in accuracy in relationship to the number of weeks post-craving assessment as craving scores increased (Fig. 2). The ratio of craving scores preceding use to craving scores preceding abstinence declined from 2.7 to 1.4 for the intervals ending one to seven weeks after the craving assessment, respectively (Fig. 3). Additionally, the closer in time to the initial assessment, the higher the relative risk of subsequent methamphetamine use for scores above the cut-off, suggesting further that the more proximal assessment yielded better estimates of the relationship between craving and methamphetamine use (Fig. 4).

AUCs below 0.70 indicated that methamphetamine craving was inaccurate at predicting methamphetamine use beyond two weeks following each craving report. Sensitivity also declined precipitously after two weeks, (48% to 26%), while the corresponding false negative rate (1-sensitivity) increased 52% to 74% the more distal the endpoint. Highest positive likelihood ratio for the most proximal assessment suggests that the potential of craving as a marker of methamphetamine use is strongest for the one-week assessment interval.

## Discussion

This longitudinal study set out to determine the potential utility of methamphetamine craving as a prognostic marker of the probability of methamphetamine use during treatment of methamphetamine dependence. Using a prospective, time-lagged, repeated-measure GLMM, within-subject design, we reproduced methods developed by Hartz and colleagues [46] and replicated their original finding that methamphetamine craving was a significant predictor of methamphetamine use in the subsequent treatment week. For internal reproducibility, we also performed double cross validation and obtained the same results. Overall, craving scores preceding use (mean = 34.9) were 2.7 times higher than scores that preceded abstinence (mean = 12.7). For the average participant, the probability of ongoing methamphetamine use increased by 0.38% for each one-point increase in craving score (0–100).

Finding that treatment and methamphetamine use the previous week also significantly predicted ongoing methamphetamine use is consistent with research demonstrating improved retention and treatment adherence in the Methamphetamine Treatment Project [64]. Nevertheless, methamphetamine craving remained a significant predictor of methamphetamine use in the subsequent treatment week after controlling for time in treatment, treatment assignment, and methamphetamine use during the prior week. Taken together, results are very similar to, and in some instances, match exactly the findings of Hartz et al. [46].

Building upon Hartz et al. we examined the strength of prediction using recently developed methods of ROC analysis for repeated measures [74] to derive an optimal cut-off point, rather than utilizing a median split cut-off. Multiple measures of predictive accuracy indicated substantial improvements compared to the initial method. GLMM estimates can be heavily biased for small samples [84], thus another advantage of this study was the large sample size. Another advantage was that GLMM allows cases with missing data points to be retained without replacement through estimation or substitution, assuming that they are randomly missing [70]. Missing data resulted in unbalance in the distribution of craving-methamphetamine use lagged pairs. In the randomly-split double cross validation, the 1/3 sample had approximately twice as many missing observations than the 2/3 sample. Yet we found the same cut-off value (27) for both samples, in effect, validating the prediction model with both a small and large number of “unknowns” [72] and suggesting further that the cut-off values were not seriously biased by missingness. ROC analyses also will establish a common metric for comparing the predictive accuracy of variables in our future studies or with variables utilized by other investigators, whether they use similar or different craving measures, methods, and designs [85].

We took the additional step of comparing the strength of prediction at one to seven week endpoints following the craving self-report (postassessment), and found a statistically significant association between craving and subsequent methamphetamine use across all time lags, though the strength of prediction was weakest seven weeks post-assessment. These findings were also consistent across different methods of analysis. For example, the relative risk and odds ratios of subsequent methamphetamine use showed the same decline in values the more distal the assessment (see Figs. 3 and 4). Sensitivity decreased from 0.47 (47%) to 0.26 (26%) at one to seven weeks after the craving report, respectively, indicating a decrease in the probability of detecting true positives the more distal the assessment. Furthermore, marked decreases in the use predictions (61% to 35%) were also observed at maximal LOWESS estimates (score = 100). Overall, predictive accuracy declined dramatically after two weeks, though predictive performance was strongest for the subsequent treatment week. Results support the need to assess methamphetamine use within one week of the craving report [46]. Similarly, alcohol, cocaine, and smoking studies have demonstrated that craving was associated with a greater likelihood of use during the subsequent treatment week [53, 54, 60, 62].

Why craving’s predictive power would diminish over time is unknown. It has been suggested that withdrawal severity declines from a high initial peak within 24 hours of the last use of methamphetamine through the first two week of abstinence [86]. Following this phase, episodes of increased methamphetamine craving may reemerge [87]. Animal models predict the reemergence of craving following intial abstinence from stimulants, but suggest further that craving does not decay, but rather increases progressively, over a two-month withdrawal period [88]. In a recent study [89], both treatment arms (assertive follow-up and coordinated care approaches for acute treatment of methamphetamine-induced psychosis) showed an increase in desire for methamphetamine during the past 24 hours at 6-months post-treatment compared to baseline measures. Studies regarding the decay of craving during the treatment of methamphetamine dependence are needed.

At the one-week endpoint, AUC suggests that methamphetamine craving was a fair predictor [76] of ongoing methamphetamine use. In a similar in-treatment population, for example, for every 100 observations, 30 (prevalence) would be true positives (actual subsequent use). With substandard sensitivity of 47%, craving would detect only 14 (hits) of those 30, leaving 16 undetected (misses). With good specificity (82%), of the 70 negatives (actual subsequent nonuse), craving would detect 57 (true negatives) and incorrectlyclassify 13 as positive (false positives). In absolute numbers, assessment would result in slightly more hits than false positive results (false alarms).

Several factors may have mitigated against the detection of true positives. Analyses may have been limited due to the high percentage of observations with craving scores equal to 0 or the low overall use rate. There may have been more actual use, but the relatively infrequent (weekly) toxicological verification used in this study may have contributed to the low prevalence of ongoing methamphetamine use. Still, the low sensitivity of craving suggests the need for improved ways to measure the immediate antecedents of actual methamphetamine use. One approach would be to measure craving and methamphetamine use more often than once per week. Electronic technology, e.g. ecological momentary assessment [90] via interactive voice response (IVR) over cellular telephones [91] could make real-time assessment both practical and advantageous. Another way to increase sensitivity without sacrificing specificity would be a parallel testing strategy, assessing simultaneously and using several valid predictors rather than one. For example, variables could be included in the prediction model to assess the predictive accuracy of craving and other variables believed to be relevant, such as stress [92], withdrawal [93], mood [94], and cue exposure [95].

Subjects also were users of alcohol, cigarettes, and marijuana in addition to methamphetamine. Concurrent use of substances other than drug of choice has been a valid predictor of ongoing use in outpatient treatment for cocaine dependence [59], while craving for one substance may be associated with increased craving for other dugs [96, 97]. A recent study [98], however, showed that most of the time methamphetamine was used alone, cigarette smoking was not associated with using methamphetamine, and neither alcohol nor marijuana increased the likelihood of ongoing methamphetamine use.

This study may also have been limited by the assessment of craving using a single item [99, 100]. Craving is commonly assessed in this manner, but a more recent trend has been the development of multifactorial scales [26], such as the Desires for Speed Questionnaire [101] and the Amphetamine Withdrawal Questionnaire [102] that could address multidimensional craving for methamphetamine. Flannery et al. [60] reported that craving as measured by multiple scales (the Penn Alcohol Craving Scale, the Alcohol Urge Questionnaire, and items 1–6 of the Obsessive Compulsive Drinking Scale) was a stronger predictor of subsequent drinking than was drinking during the prior week. When comparing 14 craving instruments, however, a single item VAS was found to be more accurate at assessing weekly fluctuations in cocaine craving than a multidimensional questionnaire [44]. A single item also is simple and efficient to use. Therefore, a single item was thought to provide an accurate assessment of craving in the present study.

In addition, the use of the term “craving” in a single-item questionnaire has previously been thought to produce an excess of false positive effects [25, 103]. But, a major finding in this study is the low number of false alarms. VAS craving also displayed consistently good specificity, such that the proportion of false alarms (false positive rates) did not increase as the number of observations increased and remained low across all assessment periods. The negative predictive value at the one week endpoint indicated further that for 79 out every 100 observations that fell below the cut-off, no actual use of methamphetamine took place. This suggests craving would be useful in a sequential screening paradigm [77], e.g. to select candidate medications for the treatment of methamphetamine disorders prior to conducting large scale clinical trials [104].

Furthermore, though predictions exhibited declines in accuracy in relationship to the number of weeks post-craving assessment as craving scores increased (Fig. 2), craving scores closer to 0 showed consistent accuracy in predicting nonuse of methamphetamine over the same endpoints. These findings suggest lower levels of craving might be more meaningful than elevated scores, which may have been mediated or moderated by factors not included in the prediction model. For example, little is known about patients’ ability to cope with these desires as time passes ([26]), including the learning of skills to manage craving and lifestyle changes that reduce the urge to use to use methamphetamine. Whether the craving assessments themselves served as a deterrent to ongoing methamphetamine use should be a topic for further study.

On the other hand, the significant findings on temporal proximity and craving suggest that some patients and clients were at short-term risk for relapse. A craving score (marker) at or above the cut-off provided some evidence for early detection (59% post-assessment probability of use), a 29% increase in predictive efficiency (cf. 30% prevalence). In this case, clinicians may decide to increase intervention intensity. In instances where the marker was below the cut-off value, clinicians might decide not to alter the treatment plan of a patient who had only a one-in-five chance (22% post-assessment probability) of methamphetamine use in the subsequent week.

Prediction, however, is not explanation. Whereas craving may predict the occurrence or nonoccurrence of methamphetamine use during treatment, the model does not help explain whether craving is a causal or contributing factor to ongoing methamphetamine use, whether use engenders craving, or both. Craving served as a surrogate for ongoing use, however, self-reported craving could be a proxy for withdrawal [10], signal drug availability [25], or reflect cognitive processes, such as conflict between the inclination to approach use and the inclination to avoid relapse [105] or planning and intention to use [106] methamphetamine. Many other more salient and temporally linked factors may also contribute to drug seeking and serve as motivational factors for methamphetamine use, for days or weeks after the craving experience. Clues have been provided by other researchers [107, 108], who have proposed that an important set of contributing factors of methamphetamine use is the unpleasant emotional and cognitive impairments that accompany the abstinence syndrome for days to months after methamphetamine use is stopped. Stress also may play a significant role in methamphetamine use [109, 110]. Another underdeveloped area is positive expectancies [111], which has been recently implicated as a key factor in methamphetamine use [112]. Having included these measures might speak to convergent or discriminant validity of the single-item craving construct in this study as well as provide a possible alternative explanation for what appears to be the influence of craving here.

Nevertheless, clinical decisions and contemporary practice are informed by systematic evidence. We found a linear time-lagged correlation between craving intensity scores and drug use, such that use followed craving independent of time in treatment or recent use of methamphetamine. Put another way, craving is a potential risk marker sufficiently distal from the endpoint to provide time to intervene and prevent ongoing drug use. Findings also suggest that the optimal window of opportunity for intervention is within one, or perhaps two, weeks. Markedly elevated craving scores also may indicate a worsened prognosis for as long as two months. We hypothesize that interventions that reduce craving will reduce subsequent methamphetamine use. Time-limited (brief), cognitive-behavioral therapy has been efficacious in improving patients’ confidence in their ability to resist craving to use methamphetamine [113]. Whether or not treatments for methamphetamine dependence are, in addition to increasing confidence about resisting craving, also able to suppress craving and thereby prevent the ensuing use of methamphetamine warrants investigation.

## Figures and Tables

**Table 1:** Demographic and Clinical Characteristics.

Age, years Mean (SD)	32.7 (8.2)
Female N (%)	355 (51.5)
Ethnicity N (%)	
Caucasian	430 (62.6)
Hispanic	81 (11.8)
Asian	48 (7.0)
Pacific Islander	45 (6.5)
Multiracial	45 (6.5)
Native American	15 (2.2)
African American	13 (1.9)
Other	10 (1.5)
Usual Route of methamphetamine administration N (%)	
Smoking	419 (61.3)
Insufflation	169 (24.7)
Injection	83 (12.1)
Oral	13 (1.9)
Days of methamphetamine use, past 30 Days Mean (SD)	12.4 (9.7)
Years of lifetime methamphetamine use Mean (SD)	5.4 (5.1)

**Table 2:** Accuracy of Craving as the Sole Predictor of Ongoing Methamphetamine Use.

	Number of weeks following craving assessment (endpoint)						
	
Summary statistic	1	2	3	4	5	6	7
*Sample characteristics*							
Number of observations (N)	2742	2342	1875	1449	1031	675	309
Number of subjects (n)	691	652	601	574	496	443	309
Prevalence of use	0.30	0.27	0.28	0.29	0.28	0.28	0.29
Prevalence of nonuse	0.70	0.73	0.72	0.71	0.72	0.72	0.71
Craving score (Mean/SD)	17/31	20/33	20/33	21/34	22/35	25/35	28/37
Craving score = 0 (%)	21	23	26	31	37	44	58
Craving score = 100 (%)	3	3	3	3	4	4	5
*Predictive accuracy*							
Area under the curve (AUC)	0.70	0.70	0.68	0.64	0.63	0.60	0.58
Optimal craving cut-off point	27	25	30	33	34	50	70
Sensitivity (Sn)	0.47	0.48	0.45	0.43	0.46	0.32	0.26
False negative (FN) rate	0.53	0.52	0.55	0.57	0.54	0.68	0.74
Specificity (Sp)	0.82	0.81	0.81	0.80	0.78	0.80	0.81
False positive (FP) rate	0.18	0.19	0.19	0.20	0.22	0.20	0.19
Positive predictive value (PPV)	0.52	0.52	0.49	0.46	0.46	0.39	0.36
Change in likelihood	0.22	0.25	0.21	0.17	0.18	0.11	0.07
Negative predictive value (NPV)	0.79	0.79	0.79	0.77	0.79	0.75	0.73
Likelihood use despite -	0.21	0.21	0.21	0.23	0.21	0.25	0.27
Change in likelihood despite -	−0.09	−0.06	−0.07	−0.06	−0.07	−0.03	−0.02
Overall correctly classified	72%	71%	71%	69%	69%	67%	65%
*Predictive efficiency*							
Negative likelihood ratio (LR−)	0.65	0.64	0.68	0.72	0.69	0.85	0.92
Positive likelihood ratio (LR+)	2.59	2.55	2.38	2.09	2.13	1.61	1.37
Pre-assessment odds use	0.43	0.37	0.39	0.41	0.39	0.39	0.41
Rule-in use (marker ≥ cut-off)							
Post-assessment odds use	1.11	0.94	0.93	0.85	0.83	0.63	0.56
Change in odds of use	0.68	0.57	0.54	0.44	0.44	0.24	0.15
Post-assessment probability use	0.59	0.49	0.46	0.46	0.45	0.39	0.36
Change in probability use	0.29	0.22	0.18	0.17	0.17	0.11	0.07
Rule-out use (marker < cut-off):							
Post-assessment odds use	0.28	0.24	0.26	0.29	0.27	0.33	0.38
Change in odds use	−0.15	−0.13	−0.13	−0.12	−0.12	−0.06	−0.03
Post-assessment probability use	0.22	0.19	0.21	0.23	0.21	0.25	0.27
Change in probability use	−0.08	−0.08	−0.07	−0.06	−0.07	−0.03	−0.02
